# Feasibility and acceptability of peer-delivered HIV self-testing and PrEP for young women in Kampala, Uganda

**DOI:** 10.1186/s12889-023-16081-0

**Published:** 2023-06-16

**Authors:** Rita Nakalega, Nelson Mukiza, Robert Menge, Samuel Kizito, Juliet Allen Babirye, Cynthia Ndikuno Kuteesa, Denis Mawanda, Emmie Mulumba, Josephine Nabukeera, Joseph Ggita, Lydia Nakanjako, Carolyne Akello, Brenda Gati Mirembe, Zubair Lukyamuzi, Catherine Nakaye, Hajira Kataike, Joel Maena, Juliane Etima, Hadijah Kalule Nabunya, Florence Biira, Christine Nagawa, Renee Heffron, Connie Celum, Monica Gandhi, Andrew Mujugira

**Affiliations:** 1 Makerere University-Johns Hopkins University (MU-JHU) Research Collaboration, Kampala, Uganda; 2 RineCynth Advisory, Kampala, Uganda; 3 University of Stavanger, Stavanger, Norway; 4 Brown School at Washington University, Saint Louis, MO USA; 5 University of Washington, Seattle, WA USA; 6 University of California San Francisco, San Francisco, California USA; 7 Infectious Diseases Institute, Makerere University, Kampala, Uganda

**Keywords:** Young women, Oral PrEP, HIV, Self-testing, Peer delivery

## Abstract

**Background:**

Adolescent girls and young women (AGYW) account for 29% of new HIV infections in Uganda despite representing just 10% of the population. Peer support improves AGYW linkage to HIV care and medication adherence. We evaluated the feasibility and acceptability of peer delivered HIV self-tests (HIVST) and oral pre-exposure prophylaxis (PrEP) to young women in Uganda.

**Methods:**

Between March and September 2021, we conducted a pilot study of 30 randomly selected young women, aged 18–24 years, who had received oral PrEP for at least three months, but had suboptimal adherence as measured by urine tenofovir testing (< 1500 ng/ml). Participants were offered daily oral PrEP and attended clinic visits three and six months after enrollment. Between clinic visits, participants were visited monthly by trained peers who delivered HIVST and PrEP. Feasibility and acceptability of peer-delivered PrEP and HIVST (intervention) were measured by comparing actual versus planned intervention delivery and product use. We conducted two focus groups with young women, and five in-depth interviews with peers and health workers to explore their experiences with intervention delivery. Qualitative data were analyzed using thematic analysis.

**Results:**

At baseline, all 30 enrolled young women (median age 20 years) accepted peer-delivered PrEP and HIVST. Peer delivery visit completion was 97% (29/30) and 93% (28/30) at three and six months, respectively. The proportion of participants with detectable tenofovir in urine was 93% (27/29) and 57% (16/28) at months three and six, respectively. Four broad themes emerged from the qualitative data: (1) Positive experiences of peer delivered HIVST and PrEP; (2) The motivating effect of peer support; (3) Perceptions of female controlled HIVST and PrEP; and (4) Multi-level barriers to HIVST and PrEP use. Overall, peer delivery motivated young women to use HIVST and PrEP and encouraged persistence on PrEP by providing non-judgmental client-friendly services and adherence support.

**Conclusion:**

Peer delivery of HIVST and oral PrEP was feasible and acceptable to this sample of young women with suboptimal PrEP adherence in Uganda. Future larger controlled studies should evaluate its effectiveness among African AGWY.

## Introduction

Adolescent girls and young women (AGYW) aged 15 to 24 years are at high risk of HIV acquisition, and accounted for 25% of new HIV infections in sub-Saharan Africa in 2021 [1]. AGYW are twice likely to be living with HIV as their male peers, and HIV-related illnesses are the leading cause of death in this population [1, 2]. The Joint United Nations Programme on HIV/AIDS (UNAIDS) estimates that AGYW in Uganda are thrice as likely to acquire HIV infection as young men [2]. In 2020, AGYW accounted for 29% of new HIV infections in Uganda despite representing just 10% of the population [2].

Behavioral change interventions for AGYW include delayed sex debut, consistent condom use and decreasing the number of sexual partners but require male partner involvement [3–6]. HIV pre-exposure prophylaxis (PrEP) is an effective prevention intervention that gives women autonomy over their HIV protection [7–9]. Oral PrEP uptake was high (~ 95%) in recent open-label PrEP studies among young African women in Eastern and Southern Africa [10–13], but PrEP persistence was low with most users dropping out of care within six months of follow-up [12, 14, 15].

HIV testing is key to PrEP delivery; testing must occur prior to initiation and is recommended quarterly to ensure that PrEP is not used during acute HIV infection when antiretroviral drug resistance could develop [8]. Strategies to reduce the length and number of PrEP clinic visits include HIV self-testing (HIVST) and incorporating peer support [16, 17]. HIVST was acceptable to 98% of PrEP users in Kenya [18], and similar findings were reported among young female sex workers in Malawi [19, 20]. HIVST reduces provider workload when delivered in overburdened healthcare systems [21], and reduces the number of clinic visits for PrEP refills without compromising adherence and HIV prevention effectiveness [22]. World Health Organization (WHO) guidance suggests peer support for HIV testing and PrEP delivery [23]. Peers work directly with participants to identify and overcome barriers and improve linkage and persistence on PrEP [24, 25]. Peer delivery of PrEP could facilitate PrEP uptake and persistence among African AGYW, but data on the real-world effectiveness of this approach are limited [16, 26]. This study aimed to assess the feasibility and acceptability of peer delivered HIVST and PrEP to young women in Uganda.

## Methods

### Study design and setting

We conducted a pilot multi-method study to evaluate the feasibility and acceptability of peer delivered HIVST and oral PrEP to young women in Kampala, Uganda from March to September 2021. The study was conducted at Kiswa Health Center III, a government-owned health facility in Nakawa division, one of five administrative divisions in Kampala. This facility offers free health services to young people at the adjacent Naguru Teenage Information and Health Centre (NTIHC), an independent youth clinic which has provided Adolescent Sexual and Reproductive Health and Rights services for approximately two decades. The youth clinic primarily targets young people aged 10–24 years in Nakawa division. Services provided include screening for HIV and treatment for other sexually transmitted infections, condom promotion and distribution, and maternal health services. All AGYW eligible for PrEP at NTIHC are linked to the adult HIV clinic at Kiswa where they are offered risk-reduction and PrEP adherence counselling according to national guidelines [27]. AGYW initiating PrEP are initially given a one-month drug supply and thereafter, PrEP is dispensed bi-monthly or quarterly, depending on personal preference. At the time of the study, about 160 women were receiving oral PrEP at this facility; approximately 60 were AGYW, of whom 40 (67%) were engaged in sex work.

### Study population, eligibility and procedures

A sample of 30 AGYW aged 18–24 years, who were on PrEP for at least one month and had suboptimal adherence, was selected from the 60 AGYW receiving PrEP at the study site using systematic random sampling. The sampling interval (2) was determined by dividing the total population (60) by the sample size (30). Duration on PrEP was determined using dispensing records at the health facility. Suboptimal adherence was defined as a negative tenofovir urine test and/or dispensing record of missing the PrEP re-fill date by > 14 days. AGYW were approached by study staff and invited to participate. At the enrollment visit, a structured questionnaire was used to collect data on participant demographics (age, level of education, monthly income, source of income, number of children), sexual behaviors, HIV risk perceptions and PrEP uptake. At the baseline, month three and month six clinic visits, HIV testing was performed by the study nurse using serial HIV rapid tests according to national guidelines [27]. Urine tenofovir testing was done using a lateral flow antibody-based immunoassay to assess recent drug exposure (over 2 days) (Abbott Rapid Diagnostics, Pomona, CA); this assay has high sensitivity (100%) and specificity (95%) [28]. The immunoassay cut-off of > 1,500 ng/mL classified participants as adherent [28]. The study nurse used drug-level feedback for real-time adherence counselling. Study implementation was guided by the framework for PrEP introduction for AGYW [29].

### Peer delivery model

Three peers aged 18–24 years were purposively selected by Kiswa Health Center staff from the AGYW receiving PrEP at the facility. The selection criteria were young women with no PrEP refill interruptions for at least one year (came to clinic and obtained PrEP bi-monthly), living within the catchment area of the study site and able to read and write. Selected peers were trained for two weeks by study staff to 1) perform and interpret HIVST, and guide clients using self-tests, and 2) support PrEP adherence through lay counselling and reminders to take pills. The study nurse provided monthly supplies of HIVST and PrEP to each peer. At enrolment, participants were assigned to a peer who provided PrEP counselling and taught them how to use HIVST kits. Participants and peers mutually identified a preferred place for delivery of prevention products, such as the young woman’s home, a neighbor’s residence, a community youth venue, or other location where AGYW received PrEP refills and HIVST kits at months one, two, four and five. AGYW were instructed to self-test prior to starting a new bottle of PrEP using the OraQuick® In-Home HIV Test (OraSure Technologies, Bethlehem, PA; sensitivity 91.7%, specificity 99.9%). At monthly visits, peers reminded participants how to use HIV self-tests, interpret results and dispose of used HIVST kits. Participants were reminded to share HIV negative results with health facility staff at month 3 and 6 clinic visits or immediately contact the clinic for confirmatory testing if results were positive. Peers provided PrEP adherence support and lay counselling (i.e., active listening, advice about side effects) during monthly visits in addition to bi-weekly telephone support.

### Qualitative data collection

Twenty randomly selected AGYW who had received peer delivered PrEP and HIVST for at least three months were invited via telephone to participate in two focus group discussions (FGD). Three peers and two health workers actively involved in the study participated in in-depth interviews (*n* = 5) [30]. All interviews and FGD were conducted by a trained social scientist (facilitator) and the corresponding author (note taker) in the participant’s preferred language; Luganda (local language) for FGD and English for IDI. The FGD and IDI guides were pilot tested among other AGYW on PrEP and health workers at a similar health facility in Kampala district. All qualitative sessions were conducted in private rooms at the study site where conversations could not be overheard. Interviews and FGD were 45 and 60 min long, respectively. All sessions were audio-recorded with participant permission and transcribed verbatim into English by the study team within one week. Quality checks were performed for each transcript, with corrections and revisions made to identified errors. Study documents (structured questionnaires, FGD guides, and consent forms) were translated from English to Luganda, back translated into English and compared with the original text. We reconciled any meaningful differences between the two versions to ensure that Luganda translations were accurate.

### Data analysis

The primary outcomes were feasibility and acceptability of peer delivered HIVST and oral PrEP. Feasibility was defined as the proportion of completed peer delivery visits. Acceptability was measured by the proportion who accepted peer-delivered PrEP and HIVST at enrollment, month 3 and month 6. PrEP adherence was estimated using the proportion with urine tenofovir levels ≥ 1500 ng/ml at the month three and six clinic visits. Data were analyzed using Stata version 14.2.2 (StataCorp, College Station, TX, USA).

We coded qualitative data using Atlas.ti, version 8.3 (Berlin, Germany) to organize the coding process [31]. During the inductive analysis, open coding was carried out to identify specific portions of text corresponding to peer delivery of HIVST and PrEP. Provisional labels were defined and illustrated to become codes, which were assembled into a codebook. Data were coded by two coders (social scientist and corresponding author) [32]. After development of the initial codebook, we reviewed the codebook for consistency of text segmentation and code application with continued inter-coder agreement. Coders reached consensus and grouped identified codes into themes after reviewing inconsistent codes. The Consolidated Criteria for Reporting Qualitative Studies checklist was used to report study findings [32].

#### Ethics approval

The study was approved by Makerere University School of Medicine Research Ethics Committee (*2020–133*) and the Uganda National Council for Science and Technology (HS845ES). Administrative clearance was obtained from Kampala Capital City Authority. Written informed consent was obtained from all participants; confidentiality and anonymity were strictly observed. Informed consent for illiterate participants was obtained in the presence of an impartial witness (guardian or other literate person not part of study team**)**. All study procedures were performed in accordance with relevant guidelines and regulations.

## Results

**Fig. 1:**
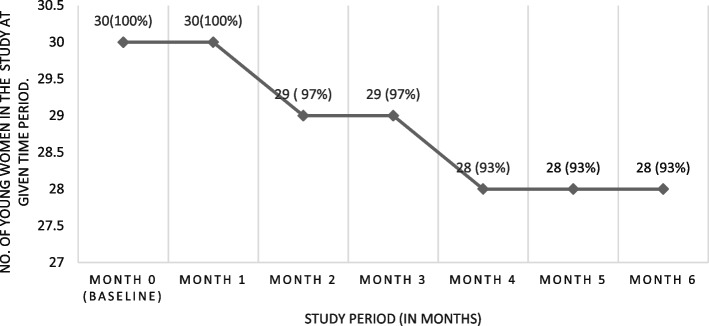
Acceptability and feasibility of peer delivered HIVST and PrEP

The median age was 20 years (interquartile range [IQR] 19–22). Of the 30 AGYW, 18 reported engaging in sex work, 17 had at least eight years of schooling, 16 had at least one child and 11 had ever been sexually assaulted (Table 1). The median monthly income was 200,000 Uganda Shillings ($56). Study retention was 97% (29/30) and 93% (28/30) at the month three and six visits, respectively. All 30 AGYW accepted to receive peer delivered HIVST and PrEP at enrollment. Acceptability and feasibility of peer delivery was 97% (29/30) and 93% (28/30) at the month three and six visits, respectively (Fig. 1). All participants performed HIVST and interpreted their results with peer support during their non-clinic visits. All self-test results were HIV-negative.

**Table 1:** Participant characteristics

Participant demographics	N (%)
**Age in years**	
18–19	14 (47%)
20–24	16 (53%)
**Has a child**	14 (47%)
**Education level**	
No schooling	1 (3%)
Primary school	12 (40%)
Secondary school	17 (57%)
**Average monthly income**	
< 100,000	10 (33%)
100,000–200,000	11 (37%)
> 200,000	9 (30%)
**Source of income**	
Sex Worker	18 (60%)
Other	12 (40%)
**Tenure status for your house**	
Owned	4 (13%)
Rented	26 (87%)
**Number of previous pregnancies**	
None	13 (43%)
One	9 (30%)
Two	8 (27%)
**Current contraceptive use (pills, implants and IUDs)**	
Yes	24 (80%)
No	6 (20%)
**Had treatment for genital symptoms in last 3 months**	
Yes	18 (60%)
No	12 (40%)
**Currently have a primary sex partner**	
Yes	25 (83%)
No	5 (17%)
**Currently married or living with primary sex partner**	
Yes	10 (33%)
No	20 (67%)
**Partnership duration**	
< 1 year	7 (23%)
1–2 years	11 (37%)
> 2 years	12 (40%)
**Primary sex partner had sex with another person in past 3 months**	
Yes	11 (37%)
No	2 (6%)
Don't know	17 (57%)
**Financial and/or material support from your primary sex partner**	
Yes	21 (84%)
No	4 (16%)
**HIV status of your primary sex partner**	
HIV negative	18 (62%)
Don't know	11 (38%)
**Condom use during the last act of vaginal sex**	
Yes	15 (50%)
No	14 (47%)
Never	1 (3%)
**No. of times you had vaginal sex in the past 30 days**	
≤ 9 times	12 (40%)
> 10 times	18 (60%)
**No. of men you had sex with in last 3 months**	
≤ 9 men	19 (63%)
> 10 men	11 (37%)

### PrEP use and HIV risk perceptions

At baseline, 26 AGYW had been on oral PrEP for ≤ 6 months and four for > 6 months. Twenty-seven experienced mild side effects which did not influence PrEP continuation. One third (10/30) reported being very worried about getting HIV in the next year, nine reported being moderately or a little worried, and 11/30 were not worried at all.

### Adherence to oral PrEP

**Fig. 2:**
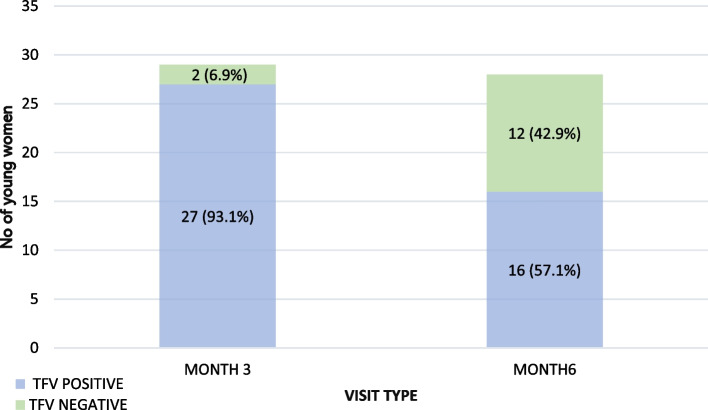
PrEP adherence during the study period. *No* Number, *TFV* Urine Tenofovir test

Tenofovir was detected in urine for 93% (27/29) and 57% (16/28) at the month three and six visits, respectively (Fig. 2). Of the 28 retained at month six, 16 were still engaged in sex work all of whom had positive urine tenofovir tests. The remaining 12 participants were not taking PrEP at levels sufficient to achieve HIV protection. Four (4/12) were pregnant, perceived themselves to be at reduced risk of HIV, reported worsening side effects because of pregnancy, and discontinued PrEP. The remaining eight were either no longer living with their partner (6/28) or had stopped sex work because of the COVID-19 pandemic lockdown (2/28) and were only taking PrEP when they anticipated sexual activity.

### Qualitative results

Of the 20 AGYW invited for FGD, five did not participate because they were busy. The remaining 15 were stratified by age: seven aged 18–19 years took part in one FGD and eight aged 20–24 years in the other. Four broad themes emerged from the qualitative data and describe the ways in which peer-delivered combination prevention was experienced by young women. The first theme describes positive experiences of peer delivered HIVST and PrEP. The second shows the motivating effect of peer support. The third describes perceptions of user controlled HIVST and PrEP. The fourth shows multi-level barriers to HIVST and PrEP use. Overall, peer delivery motivated young women to use HIVST and PrEP by providing non-judgmental client-friendly services and peer support for persistence on PrEP and adherence while enabling them to navigate and circumvent healthcare barriers to HIV prevention services.

### Theme 1. Experiences of peer delivered HIVST and PrEP

#### Convenience of peer-delivered services

Peer-delivered HIVST and PrEP was acceptable to AGYW because peers were friendly and approachable. Young women reported that they liked having peers deliver HIVST and PrEP to them because it was convenient and helped avoid transportation costs to clinic and time spent in long queues. AGYW also felt peers had more time and flexibility to attend to their needs than busy healthcare workers. Those who could not take time off work were able to receive PrEP refills at a mutually convenient place. The psychosocial support and supportive environment provided by peers, including encouragement to adhere to PrEP, was valued by young women.

“Having a peer deliver my drugs helped me because I may be at work and have no time to come to the health facility to pick the drugs [PrEP]. I may be new at the job and fear to go away from the place of work because maybe they told me that I am to be sacked anytime I am found not at my job. But I can call my peer on phone and direct her where I am and tell her to come there, attend to me and I continue working”. (AGYW, age 18)

“My peer gives me time and brings me the things [HIVST and PrEP], even if I am far, she can tell me that we meet at such and such a place and then she gives me two or three. … I call her on phone or physically go to her because I know where she stays and where she works”. (AGYW, age 20)

#### Confidentiality of peer delivery

Young women valued the privacy and confidentiality of peer delivered HIVST and PrEP, because it helped them avoid stigmatizing encounters at the clinic. They experienced judgmental attitudes and interpersonal stigma from older women at the HIV clinic, who assumed that taking PrEP implied having multiple partners in a culture where pre-marital sex is discouraged. Young women feared inadvertent disclosure of HIV test results at the crowded public HIV clinic. Confidentially was not assured because conversations could be overheard, and they could be seen by members of their communities. The desire for non-disclosure of PrEP use and HIV status motivated uptake and use of peer delivered services.

“*You would be in fear wondering whether your test results would be told to other people at the health center. But here, when the peer gives me the HIVST kit and I test myself, I get the confidence that results are for me alone. I have done the testing myself and no one else has seen me and I have got to know my status. So, personally this has pleased me*”. (AGYW, age 21)

*“At the health center, the health worker may say, ‘That one has many sexual partners and that is the reason why she is getting [PrEP]’. But if your peer is the one who brings PrEP, it is difficult for her to tell other people that you are used by many men for sex. But a health worker can even ask, ‘Why are you going to take PrEP? What has led you to doing this and sometimes refuse to give you PrEP’”.* (AGYW, age 20)

### Theme 2. The motivating effect of peer support

#### Engagement in care

The experience of peer support motivated study participation, status disclosure, and product use by young women. Peers were perceived to be approachable and friendlier than older healthcare workers. Peers were better acquainted with the challenges faced by their fellow women. Thus, it was easier for young women to relate to their peers and disclose sensitive information to them. The participant-peer relationship was perceived to be equitable unlike unequal power relationships with authoritative health workers. Nurses concurred that peers were more familiar with the sexual health experiences of AGYW. Thus, they were able to act as intermediaries between providers and participants and help AGYW navigate the healthcare system.

“What I prefer is peer-delivered services because sometimes you can find a health worker and you fear her because of her appearance, and you fail to open up to her. But with my peer, we [are] the same age and so can share with her whatever I want. I may find a health worker who is older, and I fear her because of her appearance and fail to tell her why I had gone to her”. (AGYW, age 18)

“These girls, ladies can easily express themselves to their peers, they even call her ‘Musawo’ [health worker], and they love her. You see that the bond with her is different from an older person because we have been delivering PrEP in the community. To retain them in care is very hard, and then they feel that gap between them and us, and so there is no complete total expression. They cannot really express their needs. You find that when they come here [health facility] when they are sick, they first explain their problems to their peers and then the peer comes and says, ‘Now this one has this [health problem]’ and then they also get the confidence ”. (Nurse, age 32).

#### PrEP adherence support

Young women received peer support for adherence through in-person meetings and over the phone. Support took the form of counseling, encouragement, and reminders to take PrEP. Peers used the communication skills and knowledge gained during their study training to remind participants to keep to their dosing schedules, and ensure PrEP was taken on time. They endeavored to deliver HIVST and PrEP on schedule so that participants would not miss doses because of late refills. Participants cited peers as an important source of adherence support. Especially helpful was being reminded not to “forget our time”. The shared responsibility for adherence motivated young women not to skip doses.

*“Peer delivery is very good because the truth is that before peer delivery, I used to miss some days without taking PrEP. But this time during peer delivery, my peer reminds me almost every day to take the drugs, she tells me, ‘Don’t forget our time.’… She even delivers to me the drugs in time*”. (AGYW, age 23)

The study nurse performed urine tenofovir testing and provided adherence counselling with real-time drug level feedback during clinic visits. This, coupled with peer support, motivated PrEP adherence.

*“I would continue taking my PrEP because my peer always reminded and encouraged me because I have very many sexual partners. I also knew that the urine test for the PrEP would be the evidence that I followed the instructions, so I took my drugs and expected a positive test which was true”. (*AGYW, a*ge 19)*

#### Peer support for HIVST and PrEP use

Peers used their training in HIVST and PrEP use to reinforce what AGYW were taught at the study clinic. Peers reported that their comprehensive training equipped them with the knowledge and skills to handle PrEP side effects. AGYW did not know about HIVST and took PrEP inconsistently prior to study entry. Peer education enabled them to freely ask questions about HIVST and PrEP, increasing their understanding of how and when to use PrEP. Peers helped demystify misconceptions about PrEP side effects which improved adherence.

“At the start, we did not know how to use the HIVST kits, but the study taught us how to use the kits. And when the peer brought us drugs every month at home, she came with HIVST because it was a must. When she brings the kit, she first shows it to you to confirm whether it is sealed because it is supposed to be new [unused]. She opens it in your presence, and it has three tools inside, and one of them looks like a straw [test device] and it is the one that you rub over the upper and lower gum and then it is put in the one that does the testing [buffer] and then it shows the results - negative or positive - and the results show within five minutes”. (AGYW, age 22)

“When my peer had just started bringing me the drugs, she told me the side effects I would get from the drugs, for example diarrhoea, nausea and headache. When I started taking the tablets, I got diarrhoea, nausea and got persistent headache. I went to my peer and explained to her the problems I had. She told me that the side effects would go away slowly and emphasized that I should not stop taking the drugs [PrEP]. Those effects stopped, and I have not experienced them again and I continued taking my drugs [PrEP]”. (AGYW, age 19)

### Theme 3. Perceptions of female controlled HIVST and PrEP

#### Self-controlled HIV prevention

Young women reported that PrEP and HIVST were HIV prevention methods they could use and control by themselves. They described experiencing challenges when negotiating male condom use and requesting male partners to test for HIV. Particularly challenging were clients who declined to use condoms or test for HIV, preferring to offer more money in exchange for condomless sex. Young women were desirous of these larger sums of money, even though condomless sex put them at risk of sexually transmitted infections. Taking PrEP provided young women with confidence that they were protected against HIV.

“Where we go [for sex work], I can find a man and he refuses to use a condom; sometimes the situations force us do certain things. There is a time I had unprotected sex with a man, and I did not know his HIV status, but I did it because I needed money; I was confident that the drug [PrEP] protected me though I did not think of other illnesses”. (AGYW, age 23)

“I use condoms, but some men come, and they don’t want to use condoms and then you tell him that you should first test for HIV… he does not allow to test for HIV and then he tells you that he is to give you much more money for sex. And because you want money, you allow to have unprotected sex with him”. (AGYW, age 21)

#### HIV risk perception

Young women considered themselves at high risk of HIV infection because they engaged in sex work. HIV self-tests enabled them to know their status and taking PrEP empowered them to remain HIV negative. Thus, sex work motivated continued use of HIVST and PrEP. Peers explained to participants that understanding their risk of HIV infection was a key driver of HIVST and PrEP use. They emphasized that PrEP adherence was important for HIV protection when having sex with partners of unknown status. Taking PrEP daily and self-testing monthly was an investment in one’s own health.

“The nature of work we do [sex work], we never know what is to happen. A peer tells you that when you take PrEP, you prevent HIV. And the HIVST kits really help me because I get to know my HIV status”. (AGYW, age 18)

“Most of them are at risk and so I tell them that if they don’t want to fall sick [with HIV], they should protect themselves by using PrEP…”. (peer, age 19)

### Theme 4. Multi-level barriers to HIVST and PrEP delivery

#### Lack of punctuality

Peers experienced delays while waiting for AGYWs who did not keep time or turn up for scheduled appointments. They recounted waiting for long hours, experiencing late cancellations, enduring adverse weather conditions, and travelling long distances to meet AGYW. These challenges diminished peer willingness and readiness to do their work. Nevertheless, it was preferable for a participant to come late than not show up at all.

“And another challenge I face is that a participant can tell you to meet at 10 o’clock, but you wait for her from 10 o’clock till 3 o’clock and you are hungry, and you can’t be rude to her, she tells you that she was sorting certain things. But such a participant is better than one who completely fails to turn up”. (Age 19, peer)

“The challenges I have faced are that sometimes it can rain. I can make an appointment with someone, go to the place where we agreed to meet and then she calls and tells me that she got some problems and then I feel bad. She may do it not because she did not want to come but because she got some problems”. (Age 23, peer)

#### Inadequate staffing

Peers experienced delays when requisitioning HIVST and PrEP supplies at the health center. Health workers were unable to attend to them in a timely manner due to extensive workload and limited staffing. These delays affected peer schedules and hampered the provision of supplies to young women. The downstream consequences of these delays included young women missing PrEP doses, loss of interest in HIVST and PrEP, and peer discouragement.

*“When we go to collect drugs [PrEP] from the health center, we are delayed, and the health worker keeps telling you to wait. Sometimes you can go early in the morning and [wait] up to noon [and] the health worker who was assigned to give us the drugs is still busy. Sometimes you are forced to go back home and decide to collect them some other day”.* (Age 19, peer)

*“We wait for long at the health center and in fact the health workers are very busy… so, you have to wait from morning and leave in the evening at around 5.00 pm”.* (Age 22, peer)

#### Frequent stock outs

Young women were concerned about the unreliable supply of HIVST kits and PrEP and did not believe that the clinic would always have sufficient stock. These fears were validated by peers who confirmed that clinic supplies were often insufficient when needed. The inconsistent availability of HIVST and PrEP supplies discouraged their consistent use, and undermined provider and peer messaging about consistent PrEP adherence for HIV protection.

“The organization came from somewhere and brought PrEP but sometimes we would go to them and find when they do not have PrEP for us”. (Age 19, FGD)

“They do not give us HIVST every month on time and for this month, they did not give us [HIVST] kits when we went for drugs; the health worker said they were out of stock. She called us after about 2 weeks to get them”. (Age 23, peer)

## Discussion

In this pilot study, peer delivery of HIVST and PrEP was feasible and acceptable to adolescent girls and young women in Uganda. All participants accepted peer-delivered services at baseline, and most were receiving peer services at study exit. Notably, all AGYW engaged in sex work were adherent to PrEP. Participants valued the privacy and confidentiality of peer-delivered services which enabled them to circumvent stigma, decrease time spent at clinic and save transportation costs. Peer support motivated study participation and PrEP adherence; young women found it easier to relate to peers than health workers, and peers alleviated their concerns about PrEP side effects. HIVST and PrEP were experienced as self-controlled prevention tools that young women used to protect themselves from HIV during sex work with clients who offered larger sums of money in exchange for condomless sex. Both participants and peers experienced interpersonal and health system barriers to HIVST and PrEP delivery including negative healthcare worker attitudes, inadequate clinic staffing and frequent stock-outs of PrEP commodities. Overall, peers leveraged their lived experiences and training to help participants overcome healthcare barriers, maintain persistence in care, and adhere to PrEP.

Few studies have evaluated peer delivered HIVST and PrEP to AGYW. A study in Uganda that assessed the feasibility and acceptability of peer-led oral HIVST distribution among young people (15–24 years) and adult men (> 25 years) found that 97% used the self-tests [33]. Other work in the Democratic Republic of the Congo found that peer delivery of HIVST was acceptable to 95% of adolescents [34]. Our findings agree with these studies and suggest that peer support may improve HIV prevention uptake, including HIVST and oral PrEP. Peer support may have motivated PrEP adherence in our study through increased PrEP knowledge, self-efficacy, social support for daily pill taking and youth-friendly communication between participants and peers [35]. Peers help address psychological barriers to HIV testing including lack of confidentiality, stigma and discrimination [36]. User-friendly interventions, confidentiality and peer motivation have been shown to increase prevention uptake in other studies [37, 38]. Our study suggests that peer navigation can help circumvent these barriers.

Peer support appeared to play a role in oral PrEP adherence in this study, but we could not establish its effectiveness without a control group. In the Empower study, peer support did not increase oral PrEP adherence among AGYW who participated in adherence clubs with peer support groups in South Africa and Tanzania, but young women found these clubs useful for building self-efficacy, sharing tips about PrEP side effects, social networking, and reducing isolation [39]. Approximately 70% of young women in the HPTN 082 study enrolled in adherence clubs suggesting high acceptability of peer support in a larger sample of AGYW [40]. Social networks are developed during adolescence and young adulthood, suggesting that AGYW are responsive to peer influence [36]. This may explain the high acceptability of peer services we observed. The WHO endorses peer support for adolescents for navigating the healthcare system and for linkage to HIV care [8]. Our findings suggest that peer-led services should be incorporated as part of differentiated PrEP delivery for AGYW.

Adherence counselling with drug-level feedback was highly acceptable to AGYW in our study, which is consistent with previous studies [13, 40]. Our finding of high adherence among AGYW engaged in sex work suggests individual risk perception, peer support and drug-feedback counselling motivated PrEP use [41, 42]. By contrast, PrEP adherence was similar among young women in South Africa and Zimbabwe receiving adherence counseling with drug-level feedback versus standard counselling in the HPTN 082 study [40]. The “3P” study found moderate adherence with all women receiving drug-level feedback but there was not significantly higher adherence among AGYW who received incentives conditioned on high drug levels [13]. Peer support could motivate PrEP persistence for young African women because decisions about PrEP use are often motivated by family and friends [43].

Young people report that negative attitudes of health workers, limited knowledge among health care workers about provision of oral PrEP to individuals at risk and long waiting times at clinics hinder access to oral PrEP [44–46]. In this pilot study, participants benefited from convenient PrEP supply at monthly visits and reduced monthly or bi-monthly costs of clinic visits, but continued to receive PrEP and HIV testing at their month 3 and 6 clinic visits and thus reported challenges with long waiting hours. Additionally, peers also experienced delays in receiving PrEP supplies from health workers and were discouraged by stock outs of HIVST kits. These health system barriers need to be addressed to ensure the success of peer-led approaches to HIV service delivery. Six-month PrEP dispensing decreases the number of clinic visits relative to quarterly refills, without affecting HIV testing, PrEP persistence [22] and should be integrated with HIVST and peer support.

## Strengths and limitations

Our study strengths include urine tenofovir testing and real-time drug-level feedback to support adherence counselling for AGYW. We conducted qualitative interviews (peers and health workers) and FGD (young women) enabling the evaluation of multiple perspectives of peer delivery and triangulation of findings using multiple approaches. The limitations of this pilot study include the small sample size and lack of power to evaluate factors associated with persistence on PrEP and adherence. A larger study assessing the cost effectiveness of peer delivered HIVST and PrEP could inform the scalability of this model. We conducted a single arm study and are unable to estimate the effectiveness of drug-level feedback for PrEP adherence support. Our study focused AGYW already on PrEP at a public HIV clinic. Nevertheless, our results offer useful insights that could inform differentiated PrEP delivery for AGYW especially those engaged in sex work in other settings. Future studies should evaluate the effectiveness of peer-delivered PrEP as part of differentiated service delivery.

## Conclusions

In conclusion, peer delivered HIVST and oral PrEP was feasible and acceptable to young women at risk of HIV infection in an urban setting in Uganda. High HIV risk perception among AGYW engaged in sex work facilitated prevention effective adherence. This model can be adapted to address multi-level barriers to HIV care and PrEP persistence among African AGYW in a larger controlled trial.

## Data Availability

The dataset used and analyzed during this study is available from the corresponding author on reasonable request.

## Abbreviations

AGYW: Adolescent girls and young women
AIDS: Acquired immunodeficiency syndrome
FGD: Focus Group Discussion
HIV: Human Immunodeficiency Virus
HIVST: HIV self-testing
IDI: In-Depth Interviews
PrEP: Pre-Exposure Prophylaxis
UNAIDS: Joint United Nations Programme on HIV/AIDS
